# The burden of diabetes and hyperglycemia in Brazil-past and present: findings from the Global Burden of Disease Study 2015

**DOI:** 10.1186/s13098-017-0216-2

**Published:** 2017-03-14

**Authors:** Bruce Bartholow Duncan, Maria Inês Schmidt, Ewerton Cousin, Maziar Moradi-Lakeh, Valéria Maria de Azeredo Passos, Elisabeth Barboza França, Fátima Marinho, Ali H. Mokdad

**Affiliations:** 1 Postgraduate Program in Epidemiology and Hospital de Clínicas de Porto Alegre, Universidade Federal do Rio Grande do Sul, Porto Alegre, RS Brazil; 2 Postgraduate Program in Epidemiology, Universidade Federal do Rio Grande do Sul, R. Ramiro Barcelos, 2600 Sala 414, Porto Alegre, RS 90035-003 Brazil; 3 Institute for Health Metrics and Evaluation, University of Washington, Seattle, WA USA; 4 Programa de Pós Graduação em Ciências do Adulto, Universidade Federal de Minas Gerais, Belo Horizonte, MG Brazil; 5 Programa de Pós Graduação em Saúde Pública, Universidade Federal de Minas Gerais, Belo Horizonte, MG Brazil; 6 Departamento de Doenças e Agravos Não Transmissíveis e Promoção da Saúde, Ministério da Saúde, Brasília, DF Brazil; 7 Preventive Medicine and Public Health Research Center, Department of Community Medicine, Iran University of Medical Sciences, Tehran, Iran

**Keywords:** Diabetes, Hyperglycemia, Mortality, Morbidity, Cost of illness

## Abstract

**Background:**

Diabetes, hyperglycemia, and their complications are a growing problem in Brazil. However, no comprehensive picture of this disease burden has yet been presented to date.

**Methods:**

We used Global Burden of Disease 2015 data to characterize diabetes prevalence, incidence and risk factors from 1990 to 2015 in Brazil. Additionally, we provide mortality, years of life lost prematurely (YLL), years of life lived with disability (YLD) and disability-adjusted life years (DALYs) lost due to diabetes, as well as similar data for chronic kidney disease (CKD) due to diabetes and, as an overall summary measure, for hyperglycemia, the latter expressed as high fasting plasma glucose (HFPG).

**Results:**

From 1990 to 2015 diabetes prevalence rose from around 3.6 to 6.1%, and YLLs, YLDs, and DALYs attributable to diabetes increased steadily. The crude diabetes death rate increased 90% while that of CKD due to diabetes more than doubled. In 2015, HFPG became Brazil’s 4th leading cause of disability, responsible for 65% of CKD, for 7.0% of all disability and for the staggering annual loss of 4,049,510 DALYs. Diabetes DALYs increased by 118.6% during the period, increasing 42% due to growth in Brazil´s population, 72.1% due to population ageing, and 4.6% due to the change in the underlying, age-standardized rate of DALY due to diabetes. Main risk factors for diabetes were high body mass index; a series of dietary factors, most notably low intake of whole grains and of nuts and seeds, and high intake of processed meats; low physical activity and tobacco use, in that order.

**Conclusions:**

Our study demonstrates that diabetes, CKD due to diabetes, and hyperglycemia produce a large and increasing burden in Brazil. These findings call for renewed efforts to control the joint epidemics of obesity and diabetes, and to develop strategies to deal with the ever-increasing burden resulting from these diseases.

**Electronic supplementary material:**

The online version of this article (doi:10.1186/s13098-017-0216-2) contains supplementary material, which is available to authorized users.

## Background

The United Nations and the World Health Organization have declared that the non-communicable diseases (NCDs) are the current main threat to the health of the world’s populations [1, 2], and have identified diabetes mellitus as one of the four main NCDs meriting attention. The majority of this burden now falls on low and middle-income countries (LMICs), a result of the fact that these countries, which house most of the world’s population, have witnessed dramatic rises in both the incidence and prevalence of diabetes over recent decades [3].

Yet the characterization of the extent and size of the burden attributable to diabetes in LMICs has been hampered for several reasons. Though diabetes can directly cause death and disability, most of its burden is distributed across its complications, most notably cardiovascular disease, requiring complex approaches to sum the damage. Additionally, national databases for disease incidence, prevalence, morbidity, including hospitalization, and mortality are incipient and incomplete in most LMICs.

Since its 2010 reports, the Global Burden of Disease Project has assumed an expanded international role in characterizing the frequency, risk factors, morbidity and mortality of diseases in countries around the world. Though estimates are frequently derived by modeling when data is missing, the GBD offers LMIC countries valuable information on their disease burden.

Brazil is a middle income country with a strong national health system [4]. It has to date engaged energetically in the WHO’s challenge to confront the NCD challenge [5], and over the past decade has improved its national health databases and implemented a series of surveys aimed to better assess NCD risk factors, prevalence and disease burden. The GBD 2015 has incorporated this more precise information into their internationally standardized approach to characterizing disease burden.

The objective of this report is to describe the current status and trends of the burden due to diabetes, to CKD due to diabetes, and to hyperglycemia in Brazil based on GBD 2015 project analyses.

## Methods

GBD 2015 includes an annual assessment covering 195 countries and territories from 1990 to present. It covers 310 diseases and injuries, 2619 sequelae, and 79 risk factors by age and sex. Detailed descriptions of methodology and approach of GBD 2015 have been published elsewhere [6–9]. We generated the estimates presented here with the Institute for Health Metrics and Evaluation GBD 2015 visualizations [10, 11].

**Fig. 1:**
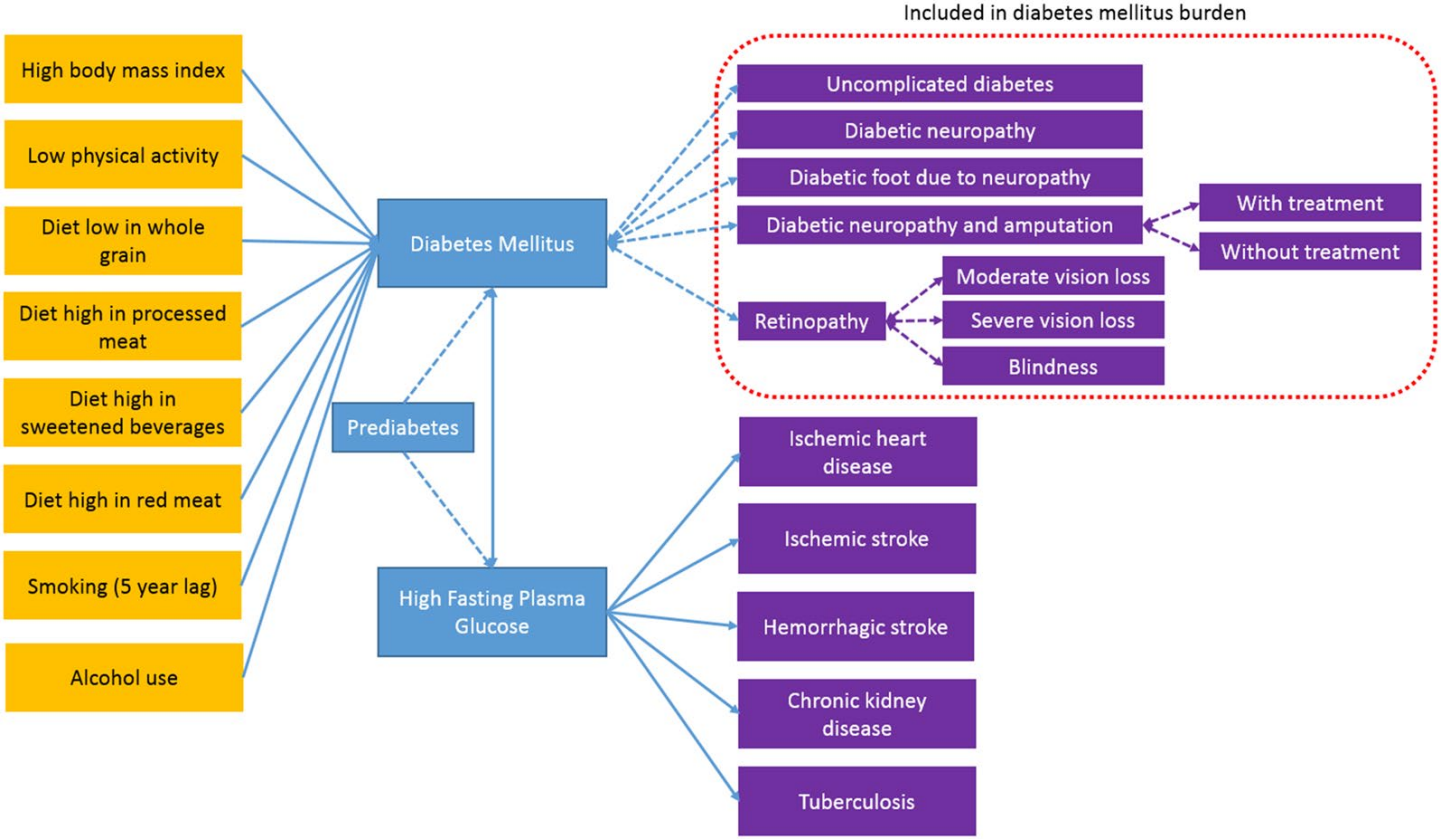
Dimensions of assessment of burden of diabetes and high fasting plasma glucose, and burden attributable to its risk factors in the Global Burden of Disease 2015 (GBD 2015 study)

The GBD 2015 framework recognizes diabetes as both a disease, with its proper outcomes, and as one of multiple causes of a series of other diseases. This latter expression of its pathology is accounted for within the risk factor category of high fasting plasma glucose (HFPG), which also encompasses the effects of lower levels of hyperglycemia. Figure 1 shows this combined burden approach. Within it, part of burden is ascribed directly to diabetes—defined through ICD-10 codes E10-13, except “0.2” codes, related to renal disease—and includes that due to living with diabetes (“uncomplicated diabetes”) and to its traditional, “microvascular” complications (vision loss, including severe low vision and blindness; neuropathy; and amputation) [12]. This direct burden is shown in the part of the Figure highlighted by the dotted red line [6]. The rest of the burden, which results from the other recognized complications of both diabetes and HFPG [13]—ischemic heart disease, ischemic stroke, hemorrhagic stroke, CKD (sub-categorized as being due to hypertension, glomerulonephritis, diabetes and other causes) and tuberculosis [7]—is accounted for through HFPG. In these latter calculations, the theoretical minimum risk exposure level of glucose was taken to be 4.8–5.4 mmol/L (86.4–97.2 mg/dL).

The GBD uses three main indicators to calculate disease burden—years of life lost due to premature mortality (YLLs), years of life lived with disability (YLDs), and the sum of the two—disability-adjusted life years (DALYs). Briefly, YLLs are calculated multiplying the number of deaths from diabetes or due to HFPG in each age group by the reference life expectancy at the average age of death for those who die in that age group [8]. YLDs were obtained by aggregating diabetes sequelae prevalences times their disability weights in age-, sex- and year-specific strata [13]. Disability weights were derived from population-based surveys of the general public [12]. All results are standardized to the world population [8].

To help understand the drivers of change in the number of DALYs due to diabetes over the period studied, following methodology previously used in the GBD project [9], we estimated percent changes due specifically to growth in total population, population ageing, and change in DALY rates. Each of these three differences is presented in absolute number of DALYs and as the relative percentage change with reference to the 1990 baseline estimate of total DALYs.

GBD 2015 identified through analysis of systematic reviews the diabetes risk factors of high body mass index; low physical activity; diets low in whole grains, nut and seeds, and fruits, and high in red and processed meat and in sweetened beverages; absence of alcohol consumption; and smoking, the latter using a time lag. The fraction of the diabetes burden attributable to each was calculated as previously described [7].

This study required no approval by a research ethics committee, as it utilized only secondary databases which are publicly available, while obeying the ethical principles of Resolution no. 466/2012 of the Brazilian Conselho Nacional de Saúde.

## Results

### Diabetes

#### Incidence and prevalence

**Fig. 2:**
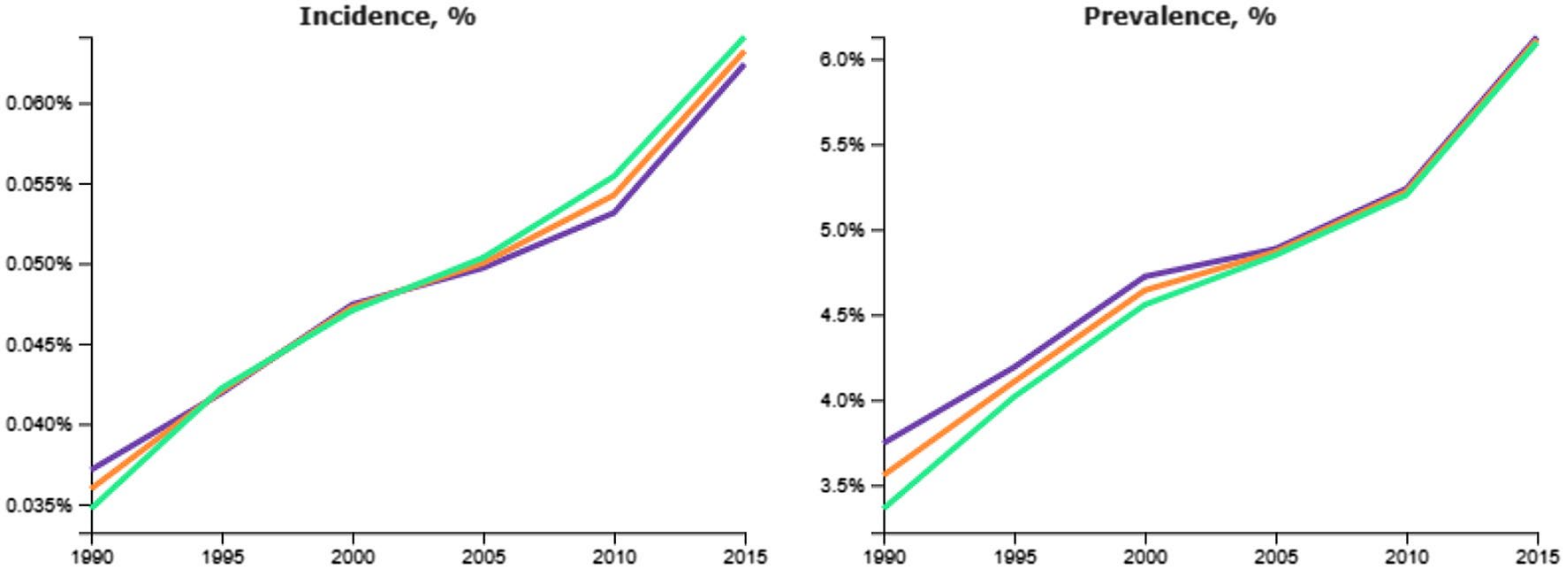
GBD 2015 estimated crude incidence (*left panel*) and prevalence (*right panel*) of diabetes in Brazil, from 1990 to 2015, standardized to the world population. *Blue line*—females, *green line*—males, *orange line*—both sexes

Figure 2 shows the prevalence (right panel) and incidence (left panel) of diabetes from 1990 to 2015, overall and separately for women and men. The prevalence rose 69% during this period, increasing from 3.6% (95% UI 3.3–3.8%) to 6.1% (95% UI 5.6–6.7%). The current prevalence is quite similar to that which GBD estimated worldwide—6.2% [11]. In 2015 approximately 12 million Brazilians had diabetes, this number growing from 2010 to 2015 by approximately 450,000 cases per year. The estimated annual incidence increased 75% over the period to a current rate of 0.63% per year. Of note, much of both of these increases in Brazil is due to population aging, as age-standardized prevalence and incidence rates have increased less—17 and 49%, respectively.

#### Burden

A total of 62,466 diabetes deaths (95% UI 59,421–65,474) occurred in 2015 in Brazil, 56.1% of them being women. This percentage was somewhat higher than the percent seen globally (50.9%) for women [6]. The crude diabetes death rate increased 90% over the period, from 15.8 (95% UI 15.4–16.3) per 100,000 in 1990 to 30.1 (95% UI 28.6–31.5) per 100,000 in 2015 (Additional file 1: Table S1). The increase in the age-standardized diabetes mortality in Brazil was much less, 4.4%, from 35.9 (34.8–37.0) to 37.5 (35.6–39.3) per 100,000.

There were 1152,332 YLLs (95% UI 1,094,056–1,206,757) due to diabetes in 2015, up 100% from the 574,910 YLLs (95% UI 558,550–593,148) in 1990. In 2015, the age-standardized YLLs rate for men was 666.7 (95% UI 622.3–709.3) per 100,000, up 3.1% from the 1990 rate of 646.7 (95% UI 619.3–676.3) per 100,000. For women, the rate was 588.0 (95% UI 550.9–625.0) per 100,000, down 17.8% from the 1990 rate of 714.9 (95% UI 685.9–747.4) per 100,000.

The total number of YLDs due to diabetes in Brazil more than doubled over the period, from 390,624 in 1990 (95% UI 269,754–39,490) to 957,912 in 2015 (95% UI 657,795–1,313,735). YLDs expanded to be responsible for 45.4% (36.6–53.1%) of all diabetes DALYs in 2015, up from 40.5% (32.1–48.4%) in 1990, a relative increase of 12%.

The crude rate of DALYs attributable to diabetes increased 58.3% over the period, from 641 DALYs (95% UI 558–741) per 100,000 in 1990 to 1015 DALYs (95% UI 865–1191) per 100,000 in 2015. In comparison, crude rate of DALYs for all diseases decreased 20.5% and the crude rate of DALYs for all other non-communicable diseases increased only 3.6% in Brazil. When age-standardized, the rate of DALYs attributable to diabetes increased by only 1% from 1990 to 2015. Table 1 shows DALY rates for diabetes for Brazil and for the world. The age-standardized DALY rates of diabetes in Brazil were 1.42 and 1.19 times of the world rates in 1990 and 2015, respectively.

**Table 1:** Age-specific, and crude (all ages) and age-standardized disability-adjusted life year (DALY) rates (/100,000) of diabetes mellitus in Brazil compared to the overall world rates, 1990 and 2015

Age group	1990						2015					
	Global			Brazil			Global			Brazil		
	Rate	95% UI		Rate	95% UI		Rate	95% UI		Rate	95% UI	
Under 5	58.9	(49.0	67.0)	62.8	(55.3	71.6)	22.1	(18.4	24.4)	24.0	(20.0	27.9)
5–9	23.0	(19.9	26.7)	29.7	(24.9	34.8)	15.7	(13.2	19.0)	15.5	(12.4	19.3)
10–14	45.1	(37.2	56.0)	67.1	(54.9	82.0)	38.0	(30.0	49.4)	39.8	(30.3	52.6)
15–19	84.0	(66.2	108.9)	116.0	(94.4	145.0)	91.6	(69.3	119.5)	89.0	(67.6	117.8)
20–24	146.9	(114.1	186.0)	170.7	(136.9	213.7)	168.7	(127.8	219.0)	143.9	(107.9	186.3)
25–29	214.0	(165.3	273.8)	249.8	(201.9	310.2)	256.8	(192.9	333.5)	224.2	(170.2	288.3)
30–34	312.2	(242.9	397.8)	358.0	(292.9	440.9)	384.1	(289.5	499.2)	331.6	(256.0	429.6)
35–39	448.2	(349.4	567.0)	523.8	(434.2	638.6)	565.0	(432.0	722.6)	495.0	(385.6	626.4)
40–44	659.4	(520.7	821.5)	799.0	(666.2	963.0)	830.7	(647.7	1053.6)	765.0	(604.4	958.9)
45–49	1022.3	(827.8	1264.5)	1246.0	(1037.0	1512.3)	1241.7	(983.4	1556.5)	1190.9	(944.3	1490.3)
50–54	1435.9	(1175.5	1747.8)	1892.2	(1591.6	2277.0)	1762.3	(1415.1	2175.0)	1820.5	(1469.0	2260.6)
55–59	1939.8	(1631.4	2336.0)	2694.1	(2292.1	3133.0)	2361.7	(1958.5	2817.1)	2581.4	(2160.5	3110.6)
60–64	2465.1	(2099.2	2906.9)	3533.7	(3072.4	4091.9)	2845.1	(2409.6	3360.3)	3514.3	(2989.2	4149.6)
65–69	2946.5	(2550.2	3462.3)	4356.6	(3836.9	4975.1)	3537.8	(3028.4	4120.4)	4461.8	(3836.4	5169.2)
70–74	3255.2	(2844.5	3752.8)	5178.5	(4684.2	5833.0)	3843.0	(3316.1	4442.0)	5292.1	(4631.1	6052.0)
75–79	3400.7	(2992.7	3889.5)	5702.8	(5189.1	6299.8)	4128.5	(3594.0	4741.4)	5976.1	(5308.4	6806.0)
80 plus	2441.4	(2166.5	2775.3)	4232.3	(3888.4	4643.9)	3185.4	(2826.0	3599.7)	5570.1	(5072.8	6144.9)
All ages (crude)	567.9	(480.5	672.2)	641.4	(558.1	740.8)	870.1	(725.7	1032.6)	1015.3	(865.3	1191.4)
Age-standardized	769.9	(654.1	907.0)	1094.2	(962.6	1247.7)	925.8	(776.1	1096.3)	1102.8	(948.7	1285.4)

As shown in Table 2, population growth accounted for an increase of 41.9%, aging of the population for an increase of 72.1%, and change in the underlying age- and sex-standardized rates of DALYs for an increase of 4.6% in the absolute number of DALYs due specifically to diabetes over the period.

**Table 2:** Decomposition analysis of the change of DALYs (thousands) due to diabetes from 1990 to 2015 as being due to total population growth, population aging, and changes in age-, sex-specific DALY rates of diabetes for Brazil

	Value	95% uncertainty interval	
1990 DALYs (thousands)	965,533	840,159	1,115,075
DALYs expected with 2015 population, 1990 population age structure, 1990 DALY rates (thousands)	1,369,996	1,192,103	1,582,182
DALYs expected with 2015 population, 2015 population age structure, 1990 DALY rates (thousands)	2,065,958	1,775,425	2,416,933
2015 DALYs (thousands)	2,110,242	1,798,473	2,476,379
Percent change from 1990 due to population growth	41.9%		
Percent change from 1990 due to population ageing	72.1%		
Percent change from 1990 due to change in DALY rates	4.6%		
Percent change from 1990 to 2015 (total)	118.6%		

**Fig. 3:**
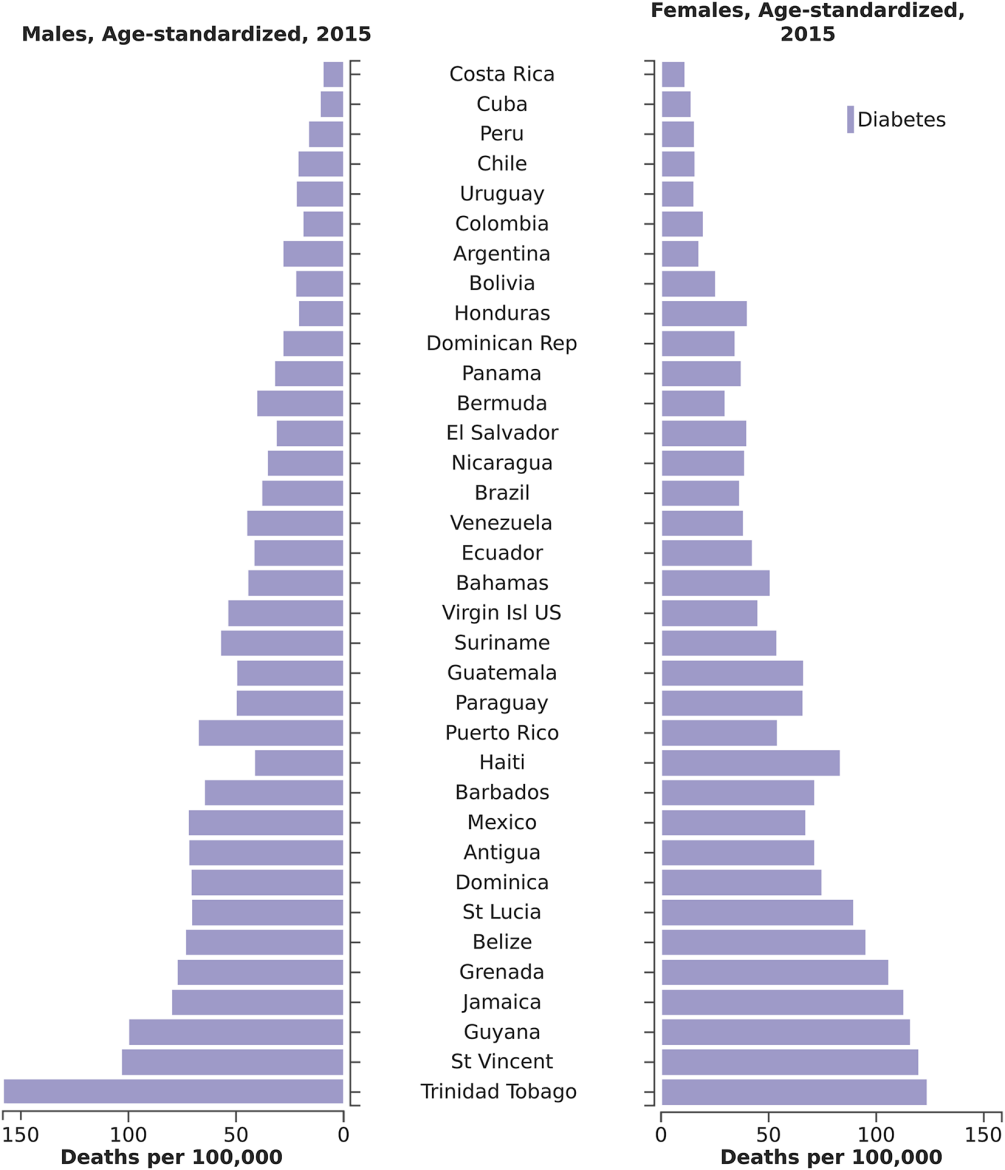
Age-standardized rates of death attributable to diabetes across countries of Latin America and The Caribbean, 2015

Figure 3 compares age-standardized rates of deaths due to diabetes across countries in the Latin American and Caribbean Region. Brazil’s rates are mid-range, although relatively high if considering only South American countries.

#### Risk factors

**Fig. 4:**
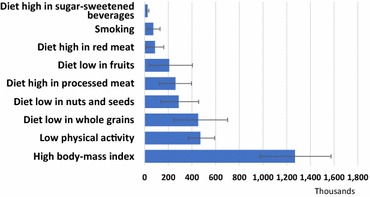
Risk factors for diabetes, expressed in terms of DALYs attributable to diabetes. 2015 Brazil

Figure 4 shows the contribution, in DALYs, of each diabetes risk factor identified by the GBD 2015. High body mass index, dietary risks (principally low whole grain and low nuts and seeds consumption, and high processed meat consumption), and low physical activity were the most important risk factors for diabetes.

Table 3 presents the population attributable fractions of diabetes DALYs and deaths due to each risk factor, as well as rates of diabetes DALYs and deaths due to these risk factors in Brazil in 2015. No significant association was found for alcohol use.

**Table 3:** Population attributable fraction of disability adjusted life years (DALYs) and deaths due to diabetes, as well as diabetes burden resulting from different risk factors in Brazil, 2015

	Population attributable fraction						Attributable diabetes burden (rate per 100,000)					
Risk factors	PAF DALYs (%)	95% UI (PAF DALYs)		PAF deaths (%)	95% UI (PAF deaths)		Rate of diabetes DALYs	95% UI DALYs rate		Rate of diabetes deaths	95% UI death rate	
High fasting plasma glucose	100			100			1015.3	865.3	1191.4	30.1	28.6	31.5
High body mass index	60.1	49.8%	69.6%	48.0	37.1%	59.5%	610.7	468.8	757.9	14.4	11.1	17.9
Low whole grains	21.4	11.8%	32.4%	17.2	9.4%	26.4%	217.3	118.2	337.2	5.2	2.8	7.9
Physical inactivity	22.3	18.1%	26.7%	23.9	19.5%	28.2%	226.5	177.8	284.5	7.2	5.9	8.5
High sweetened beverages	1.2	0.8%	1.7%	0.9	0.6%	1.2%	11.9	7.8	17.6	0.3	0.2	0.4
Low nuts and seeds	13.6	7.2%	20.9%	10.9	5.7%	17.0%	138.1	66.2	219.5	3.3	1.7	5.1
High red meat	4.1	0.5%	7.5%	3.0	0.4%	5.4%	42.0	5.0	77.9	0.9	0.1	1.7
Smoking	3.5	1.1%	6.1%	2.8	0.9%	5.0%	35.0	11.1	62.7	0.9	0.3	1.5
High processed meat	12.3	6.1%	18.0%	9.5	4.7%	13.8%	125.3	59.9	190.5	2.9	1.4	4.2

*PAF* population attributable fraction

### Chronic kidney disease related to diabetes

The GBD disease category of CKD due to diabetes mellitus permits a specific evaluation of this aspect of the diabetes burden. In 2015, 21,519 individuals (95% UI 19,906–24,481) died from CKD due to diabetes in Brazil. Between 1990 and 2015, crude CKD death rates due to diabetes increased from 4.64 (95% UI 4.30–5.37) per 100,000 to 10.35 (95% UI 9.58–11.78) deaths per 100,000. In 2015, the age-standardized death rate of CKD due to diabetes was 12.45 per 100,000 (95% UI 11.52–14.08) in Brazil, approximately 2 times the global rate of 6.50 per 100,000 (95% UI 6.06–6.86).

Considering not just deaths but also disability, and excluding the effect of population growth, the burden of CKD due to diabetes increased more than 50% over the period studied, as the crude rate of DALYs increased from 158 (95% UI 144–176) per 100,000 in 1990, to 261 (95% UI 239–298) per 100,000 in 2015. The age-standardized DALY rate per 100,000 was 281 (95% UI 258–320) in Brazil in 2015, up 11% from 1990 and almost double the overall world rate of 163 (95% UI 150–177). In 2015, 49.5% of the overall CKD burden was due specifically to diabetes in Brazil, up from 38.8% in 1990.

### High fasting plasma glucose (HFPG)

**Fig. 5:**
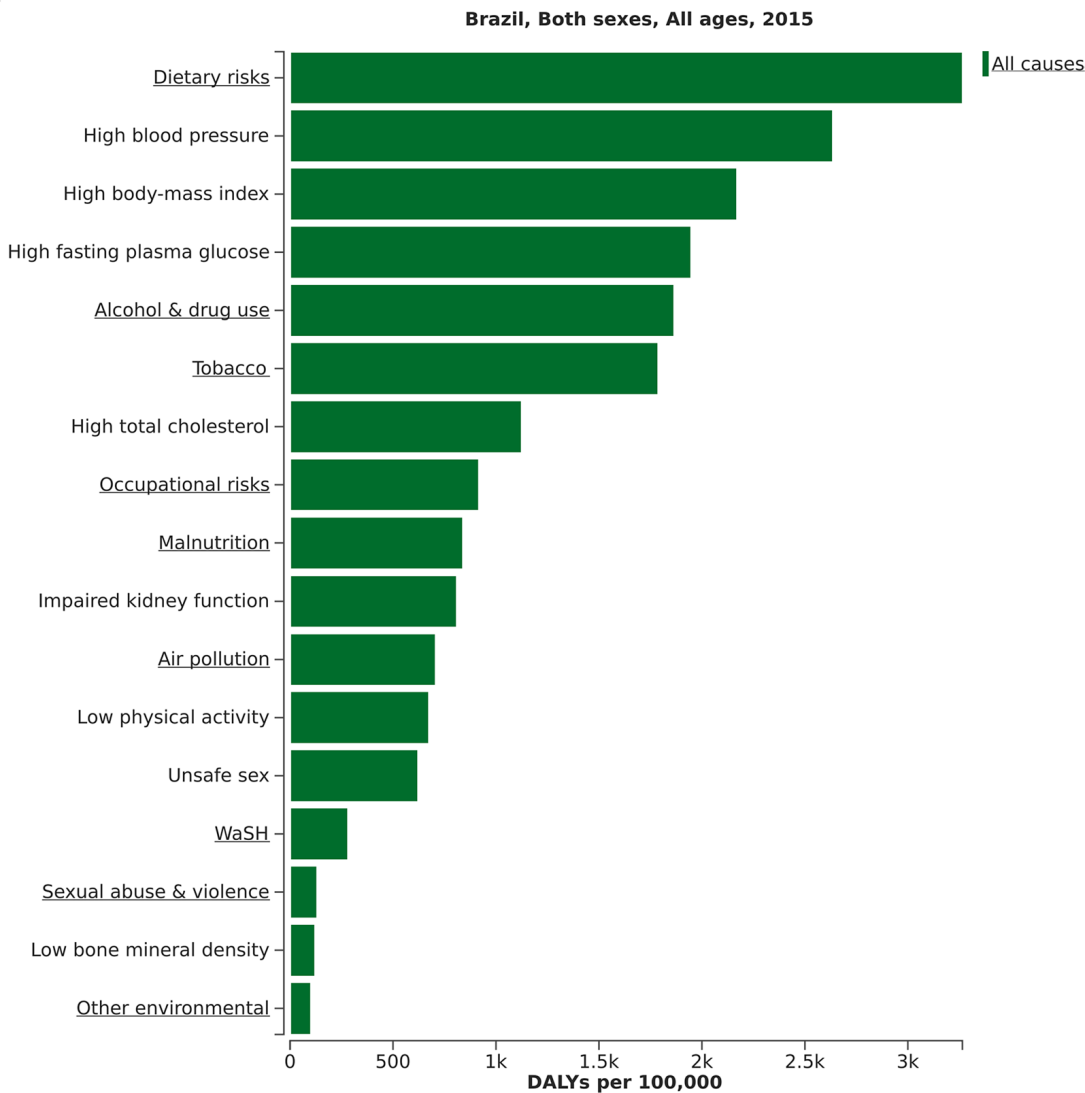
Relative importance of high fasting plasma glucose within the context of all risk factors identified by the GBD 2015. Brazil, 2015. *WaSH* water, sanitation and hygiene

As mentioned above, only part of the DALYs resulting from diabetes occurs through living with diabetes or suffering its traditional microvascular complications. We thus next explored the broader burden which includes that attributable via other diseases for which diabetes and intermediate hyperglycemia are considered causal, identified in GBD 2015 as ischemic heart disease, stroke, chronic renal disease and tuberculosis. Figure 5 shows the relative size of this burden, when expressed by HFPG, in relation to that of other risk factors. In 2015, HFPG was the 4th leading cause of overall DALYs in Brazil.

**Fig. 6:**
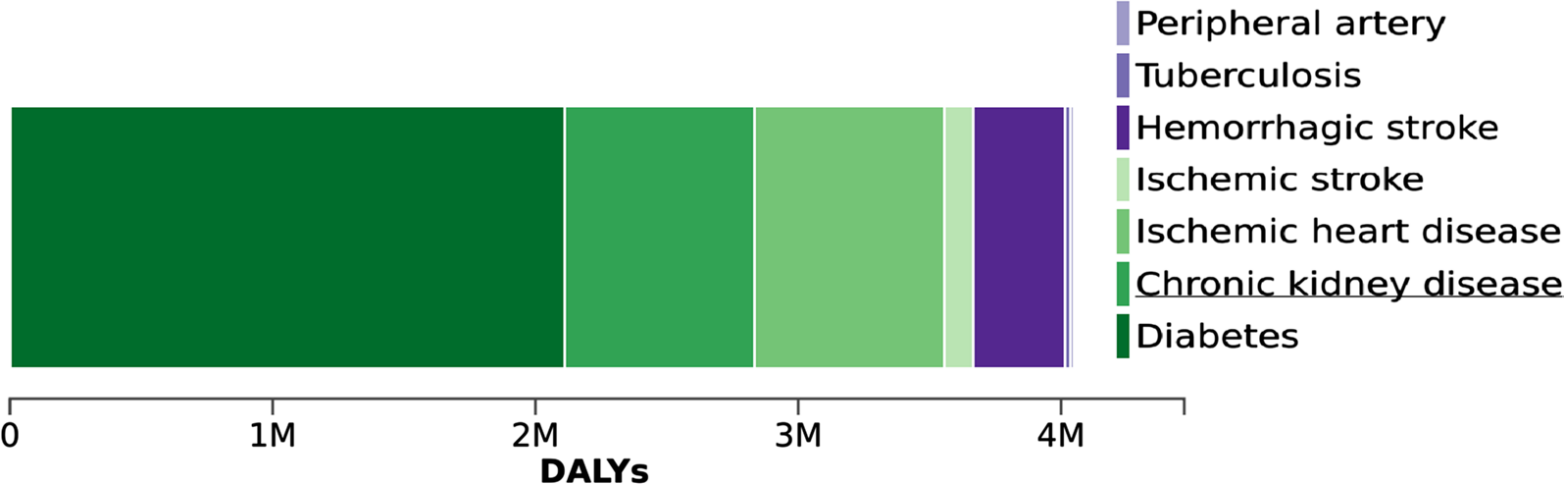
Distribution of disease causes of DALYs attributable to high fasting plasma glucose. Brazil, 2015

The attributable burden to HFPG was 1441 DALYs per 100,000 (95% UI 1276–1633) in 1990, which increased by 35.2% to 1686 DALYs per 100,000 (95% UI 1339–2140) in 2015. In 2015, 52.1% of these DALYs arose directly from difficulties related to living with diabetes or to suffering direct diabetes complications, as detailed in Fig. 1; 17.9% from ischemic heart disease; 17.8% from chronic kidney disease; 8.6% from hemorrhagic stroke; 0.5% from tuberculosis; 2.7% from ischemic stroke; and 0.3% from peripheral artery disease (Fig. 6).

HFPG was responsible (PAF) for 65.9% (95% UI 61.0–71.1) of all CKD DALYs, 18.7% (95% UI 13.0–25.4) of all ischemic heart disease DALYs, 10.2% (95% UI 6.7–14.0) of all tuberculosis DALYs, 17.6% (95% UI 12.1–24.6) of all hemorrhagic stroke DALYs, 17.2% (95% UI 10.3–29.1) of all ischemic stroke DALYs, and 26.6% (95% UI 23.7–29.8) of all peripheral artery disease DALYs.

DALYs due to HFPG represented 4.1% of all DALYs and 8.2% of NCD DALYs in 1990. By 2015, with a total of 4,049,510 DALYs estimated to be lost due to diabetes or HFPG, these relative burdens had climbed to 7.0 and 9.8%, respectively.

## Discussion

This report, to our knowledge, is the first comprehensive evaluation of the large and growing burden of diabetes and HFPG, including CKD, in Brazil. From 1990 to 2015 Brazil, like most of the world´s countries, witnessed major improvements in health, so that, despite population aging, the annual crude rate of DALYs, considering all diseases, decreased. In stark contrast, DALYs due to diabetes and HFPG climbed considerably. As a result, the fraction of total DALYs due to diabetes and HFPG increased 70%. Given the relatively greater increase in YLDs than in YLLs, this burden is now relatively evenly distributed between mortality and morbidity. The burden of CKD due to diabetes grew at an even more rapid pace.

The magnitude of Brazil’s diabetes burden places it mid-range in the ranking of Latin American countries. However, this fact should provide little comfort as the Latin American region is particularly affected by the combined obesity and diabetes epidemics [7].

The huge burden of diabetes and HFPG in Brazil has received less attention than it merits due to several possible reasons. First, the upward shift in the prevalence of self-reported diabetes has never been dramatic from one year to the next, rather following a slow but steady rise. Second, the prevalence of diabetes, as usually reported, for example 6.2% in Brazil’s nationally representative sample in 2013 [14], is based on self-report, which accounts for only part of the total cases of diabetes, since an important fraction of cases are undiagnosed. Data from ELSA-Brasil suggest that when a full accounting of diabetes is performed, including not only self-reported information on a previous diagnosis or use of anti-diabetic medication but also measurements of plasma glucose (at fasting and 2 h post load) and glycated hemoglobin, the prevalence increases by 50–100%, depending on the laboratory measurements considered. Third, prevalence estimates based on the whole population (the GBD approach) or the whole adult population (as in VIGITEL and in nationally representative Brazilian surveys) do not adequately illustrate cumulative risk across the lifespan, in that few individuals are affected during childhood, adolescence and young adulthood. In the ELSA-Brasil cohort, the prevalence of diabetes above age 60 was over 30% [15]. Thus, the lifetime prevalence in ELSA participants, which is probably a low estimate for all Brazilians given the relatively high educational level of this cohort, is at least 30%. We believe that the recent near doubling of self-reported prevalence combined with this current estimate of lifetime prevalence provide a better vision of the current and probable future size of the diabetes epidemic. Fourth, as the complications of diabetes usually take years to develop, and the epidemic is advancing, the current picture very likely underestimates to a large extent the future diabetes burden.

Great attention in Brazil has focused recently on the epidemic infectious diseases Zika, dengue and chikungunya. While these diseases are important, and the extent of the chronic burden of Zika is yet to be fully known, it is hard to imagine that their impact will come close to that currently resulting from diabetes. For example, as of June, 2016, the Ministry of Health had identified approximately 1600 cases of microcephaly, most not confirmed as being due to Zika [16]. This contrasts with the GBD 2015 estimates of approximately 12 million Brazilians currently having diabetes, over 450,000 new cases of diabetes per year, and the staggering total annual loss of 4,049,510 disability adjusted years of life due to diabetes and hyperglycemia.

In 2011, the Brazilian government developed a comprehensive strategy to confront the NCDs. Available indicators show that this strategy is in general advancing well in terms of risk factor levels, disease prevalence and mortality [17]. The rapidly rising prevalences of obesity and diabetes are the glaring exceptions. An additional issue is that the problems caused by obesity and diabetes go far beyond health. Diabetes, along with other major NCDs, has been recognized to impact negatively in a large and growing manner on the economic welfare of nations. If not adequately addressed, the cost involved in caring for diabetes and its complications along with the loss in productivity resulting from the disease could significantly hinder future economic development [18].

Controlling the diabetes epidemic will require attention to its causes. Though these causes are not yet completely understood, the known driving forces, in addition to growth and aging of the population, are mainly related to the concomitant obesity epidemic. Figure 5 shows that a high body mass index to be the 3rd leading risk factor for overall disease burden in Brazil.

Our findings thus call for increased efforts to control diabetes and its complications especially CKD, through adequate health promotion, prevention, early detection, and medical care. Obesity and diabetes, like all diseases, are socially determined, and as such require not just individual actions but socially constructed solutions. Recent efforts led by the Ministry of Health in Brazil, such as the new nutritional guidelines [19], and efforts at all levels of government to stimulate greater physical activity in leisure e and in commuting to and from the workplace merit support and need to be expanded. As many prevention initiatives with major impact on diabetes rely on policies that are implemented outside the health sector, e.g. regulation of food products, investments to increase access to leisure and physical activities, public safety policies and taxes on tobacco products, focus on coordinated efforts across multiple sectors, such as that which has been led by CAISAN in recent years to meet the challenge of obesity [20, 21], are critical. The presence of several factors not traditionally considered as conferring risk for diabetes—low consumption of whole grains and seeds and nuts, and high consumption of red and processed meat—emphasize the importance of incorporating new evidence into public health and clinical actions.

Additionally, diabetes is the quintessential life course disease, as its risk factors and its underlying pathophysiology of increased insulin resistance followed by decreased insulin secretion have been shown to span across the life cycle. In this regard, a nationally representative survey of 9th grade Brazilian school children showed a greater frequency of intake of sugar sweetened beverages than of fruits, and a greater frequency of watching television ≥2 h/day than of participating in physical education classes ≥2×/week. Fully 17.8% (17.1–18.4%) of these adolescents considering themselves to be obese [22]. Additionally, a recent representative survey conducted in the nation’s capital cities showed that only 8% of Brazilian adults aged 18–29 were pursuing a healthy lifestyle when that was defined as not smoking, undertaking adequate leisure time physical activity, and eating fruits and vegetables at least 5 days a week [23]. If healthier lifestyles in childhood and young adulthood cannot be stimulated, there is little hope for adequate control of obesity and diabetes in later life.

Health care systems, both the public Sistema Único de Saúde and those of the private supplementary health care sector, must also be adapted, for example, through the chronic care model [24], to work effectively to prevent and manage obesity, diabetes, and their complications. Data from ELSA-Brasil suggest that for every individual with diabetes, at least one additional one will have intermediate hyperglycemia [15]. The level of hyperglycemia of many of these places them at greater than 40% risk to progress to diabetes within the next decade [25]. Repeated clinical trials have shown diabetes can be prevented or at least significantly delayed with lifestyle interventions in up to 50% of those with impaired glucose tolerance [26]; and that these interventions can be translated into general practice, though with somewhat less effectiveness [27]. In this regard, the Family Health Strategy, with its emphasis on team care, including community health workers with their special role of delivering culturally appropriate health education, and its greater presence in socio-economically less favored settings where the prevalence and consequences of hyperglycemia are greatest, should be a key to confronting the obesity and diabetes epidemics at the level of the individual.

Much benefit can also be gained by the adequate treatment of those with diabetes. The evidence base for the prevention of diabetes complications is well established. Within these efforts, additional focus needs to be given to the prevention of CKD among those with diabetes. With the major increase in prevalence of diabetes, CKD due to diabetes has expanded rapidly. Though not all CKD in diabetes is due to diabetes, those with diabetes are an easily identifiable sub-group of adults at greatly increased risk, and effective clinical strategies exist to retard the advance of this disease [28]. That rates of DALYs resulting from CKD due to diabetes in Brazil in 2013 were approximately twice overall world rates emphasizes the gains in avoidable health care costs that could be reaped with better prevention of this condition.

The stability in mortality over time shown here, in the face of an ever-increasing number of individuals with diabetes, means that mortality among those with diabetes is declining, probably due in large part to improvements in health care over recent decades. As a result, afflicted individuals will live longer, which additionally increases prevalence and thus adds to the economic burden of the disease. In this regard, recent estimates suggest that men and women currently with diabetes in the US will live, on average, 28 years with their disease [29]. Thus, even if we are successful in decreasing the incidence of diabetes and death from its complications, prevalence and continued disease burden will continue to accrue from the greater fraction of the population who will suffer YLDs from decades of exposure to diabetes-level hyperglycemia.

Strengths of this report stem from the extensive data collection and innovative data analysis techniques developed by the Institute for Health Metrics and Evaluation for use in the GBD project. These permit estimations in the presence of data that are limited in extent and of less than optimum quality.

Aside from the limitations due to uncertainties with respect to the prevalence of diabetes and hyperglycemia, rates of progression to and excess mortality from complications, and disability weights of diabetes sequelae in Brazil, an additional major limitation of this study merits discussion. GBD 2015 does not take into account the more recently recognized complications of diabetes in calculations of DALYs. Approximately 40% of diabetes deaths are now estimated to be non-vascular, with diabetic renal disease representing only a small fraction of these deaths. Important non-vascular complications include liver, pancreas, ovary, colorectal, lung and breast cancers; pneumonia and other infections, chronic obstructive pulmonary disease, and a broad range of other medical conditions [30]. Thus, the overall burden of diabetes and intermediate hyperglycemia presented here may be seriously underestimated. Finally, we recognize the difficulty in using mortality data to characterize diabetes burden. The GBD approach, like most, is based on underlying cause of death, and diabetes has a strong role not only as an underlying cause but also as a contributory cause. In this regard, one of the main advantages of the GBD approach is the additional analyses of burden based on high fasting plasma glucose, which take into account the burden of diabetes and intermediate hyperglycemia as contributory causes.

## Conclusions

In conclusion, diabetes and hyperglycemia in general play a large and rapidly growing role in the burden of disease in Brazil. Health authorities and society in general would do well to pay greater attention to the prevention and management of these conditions.

## Additional file

**Additional file 1.** Age-sex specific death rates of diabetes mellitus (per 100,000 population) in Brazil, 1990 and 2015.

## Abbreviations

CKD: chronic kidney disease
DALYs: disability-adjusted life years
GBD: Global Burden of Disease
HFPG: high fasting plasma glucose
LMICs: low and middle income countries
NCDs: non-communicable diseases
YLLs: years of life lost prematurely
YLDs: years of life lived with disability
95%UI: 95% uncertainty interval
