# Supplementary material for: The burden of diabetes and hyperglycemia in Brazil-past and present: findings from the Global Burden of Disease Study 2015
Source: Diabetol Metab Syndr. 2017 Mar 14;9:18. doi: 10.1186/s13098-017-0216-2 (PMC5348777; doi:10.1186/s13098-017-0216-2)
Supplement: Supplementary file 1 — Additional file 1. Age-sex specific death rates of diabetes mellitus (per 100,000 population) in Brazil, 1990 and 2015. [file 13098_2017_216_MOESM1_ESM.docx]

**Supplementary Materials**

Supplementary Table 1. Age-sex specific death rates of diabetes mellitus (per 100,000 population) in Brazil, 1990 and 2015

| Age Group | Male | | | | | |  | Female | | | | | | |  | | Both Sexes | | | | | | | | | |  |
| --- | --- | --- | --- | --- | --- | --- | --- | --- | --- | --- | --- | --- | --- | --- | --- | --- | --- | --- | --- | --- | --- | --- | --- | --- | --- | --- | --- |
|  | 1990 | | | 2015 | | |  | 1990 | | | 2015 | | | |  | | 1990 | | | | | 2015 | | | | |  |
|  | Rate | 95% UI | | Rate | 95% UI | |  | Rate | 95% UI | | | Rate | 95% UI | | |  | | Rate | | 95% UI | | | Rate | | 95% UI | | |
| Under 5 | 0.8 | 0.7 | 0.9 | 0.3 | 0.2 | 0.4 |  | 0.6 | 0.5 | 0.8 | 0.2 | | 0.2 | 0.3 |  | | 0.7 | | 0.6 | | 0.8 | 0.3 | | 0.2 | | 0.3 |  |
| 5 to 9 | 0.2 | 0.2 | 0.3 | 0.1 | 0.1 | 0.1 |  | 0.3 | 0.3 | 0.3 | 0.1 | | 0.1 | 0.2 |  | | 0.3 | | 0.2 | | 0.3 | 0.1 | | 0.1 | | 0.1 |  |
| 10 to 14 | 0.3 | 0.3 | 0.4 | 0.2 | 0.1 | 0.2 |  | 0.7 | 0.6 | 0.8 | 0.3 | | 0.2 | 0.4 |  | | 0.5 | | 0.4 | | 0.6 | 0.2 | | 0.2 | | 0.3 |  |
| 15 to 19 | 0.5 | 0.5 | 0.6 | 0.4 | 0.3 | 0.5 |  | 1.1 | 1.0 | 1.2 | 0.6 | | 0.6 | 0.7 |  | | 0.8 | | 0.8 | | 0.9 | 0.5 | | 0.5 | | 0.6 |  |
| 20 to 24 | 1.1 | 1.0 | 1.2 | 0.8 | 0.7 | 0.9 |  | 1.4 | 1.3 | 1.5 | 0.8 | | 0.7 | 0.9 |  | | 1.2 | | 1.1 | | 1.3 | 0.8 | | 0.7 | | 0.9 |  |
| 25 to 29 | 2.0 | 1.8 | 2.2 | 1.5 | 1.3 | 1.7 |  | 1.8 | 1.7 | 2.0 | 1.2 | | 1.0 | 1.3 |  | | 1.9 | | 1.8 | | 2.1 | 1.3 | | 1.2 | | 1.4 |  |
| 30 to 34 | 3.5 | 3.2 | 3.9 | 2.6 | 2.3 | 2.9 |  | 2.6 | 2.3 | 2.9 | 1.7 | | 1.5 | 1.8 |  | | 3.1 | | 2.8 | | 3.3 | 2.1 | | 1.9 | | 2.3 |  |
| 35 to 39 | 6.0 | 5.4 | 6.6 | 4.4 | 3.9 | 5.0 |  | 4.1 | 3.7 | 4.5 | 2.7 | | 2.4 | 3.0 |  | | 5.0 | | 4.7 | | 5.4 | 3.5 | | 3.3 | | 3.9 |  |
| 40 to 44 | 10.1 | 9.2 | 11.1 | 8.0 | 7.2 | 8.9 |  | 7.7 | 7.1 | 8.4 | 5.3 | | 4.7 | 5.9 |  | | 8.9 | | 8.3 | | 9.5 | 6.6 | | 6.1 | | 7.2 |  |
| 45 to 49 | 17.0 | 15.5 | 18.5 | 14.3 | 12.9 | 16.0 |  | 15.1 | 13.9 | 16.6 | 10.4 | | 9.4 | 11.6 |  | | 16.0 | | 15.0 | | 17.1 | 12.3 | | 11.4 | | 13.3 |  |
| 50 to 54 | 29.1 | 26.7 | 31.9 | 27.4 | 24.7 | 30.4 |  | 29.2 | 26.9 | 31.8 | 20.7 | | 18.6 | 22.7 |  | | 29.2 | | 27.4 | | 31.0 | 23.9 | | 22.1 | | 25.8 |  |
| 55 to 59 | 48.2 | 44.4 | 52.7 | 47.5 | 42.8 | 52.4 |  | 55.2 | 50.6 | 60.1 | 39.7 | | 35.7 | 43.8 |  | | 51.9 | | 49.0 | | 55.0 | 43.4 | | 40.1 | | 46.3 |  |
| 60 to 64 | 75.9 | 70.2 | 82.4 | 82.4 | 74.9 | 90.4 |  | 93.2 | 85.6 | 101.6 | 71.6 | | 65.2 | 79.3 |  | | 85.1 | | 80.1 | | 90.4 | 76.6 | | 71.3 | | 82.1 |  |
| 65 to 69 | 120.6 | 112.0 | 129.6 | 132.2 | 119.8 | 145.3 |  | 146.8 | 135.9 | 158.4 | 123.2 | | 112.1 | 134.9 |  | | 134.9 | | 127.6 | | 143.1 | 127.3 | | 118.3 | | 136.2 |  |
| 70 to 74 | 186.3 | 173.2 | 201.3 | 201.9 | 184.1 | 222.4 |  | 231.8 | 214.5 | 250.2 | 195.8 | | 179.0 | 212.4 |  | | 212.2 | | 200.2 | | 224.2 | 198.5 | | 186.2 | | 212.6 |  |
| 75 to 79 | 282.2 | 259.7 | 304.8 | 301.4 | 275.1 | 330.9 |  | 335.0 | 312.5 | 360.8 | 304.9 | | 278.7 | 332.5 |  | | 313.9 | | 297.7 | | 331.2 | 303.4 | | 283.7 | | 323.6 |  |
| 80 plus | 405.5 | 377.7 | 433.1 | 615.2 | 560.3 | 672.0 |  | 473.9 | 439.9 | 509.8 | 650.2 | | 593.7 | 703.4 |  | | 449.6 | | 424.2 | | 475.5 | 637.4 | | 590.6 | | 682.0 |  |
| All Ages | 13.1 | 12.5 | 13.7 | 26.8 | 25.0 | 28.6 |  | 18.5 | 17.7 | 19.3 | 33.2 | | 31.1 | 35.2 |  | | 15.8 | | 15.4 | | 16.3 | 30.1 | | 28.6 | | 31.5 |  |
| Age-Std* | 32.8 | 31.3 | 34.2 | 38.1 | 35.6 | 40.6 |  | 38.0 | 36.4 | 39.7 | 36.7 | | 34.4 | 39.0 |  | | 35.9 | | 34.8 | | 37.0 | 37.5 | | 35.6 | | 39.3 |  |

UI = Uncertainty interval

* Age-standardized
